# Exploring the temporal relationship between mood, alcohol- and nicotine use in bipolar disorder using time-series analyses

**DOI:** 10.1186/s40345-025-00388-5

**Published:** 2025-05-23

**Authors:** Stine Holmstul Glastad, Ole Klungsøyr, Sofie Ragnhild Aminoff, Roger Hagen, Thomas Bjella, Magnus Johan Engen, Siv Hege Lyngstad, Cecilie Busch, Romain Icick, Bruno Etain, Ingrid Melle, Ole A. Andreassen, Margrethe Collier Høegh, Trine Vik Lagerberg

**Affiliations:** 1 Section for Clinical Psychosis Research, Division of Mental Health and Addiction, Oslo University Hospital, Oslo, Norway; 2 Department for Research and Innovation, Oslo Centre for Biostatistics and Epidemiology, Oslo University Hospital, Oslo, Norway; 3 Department for Research and Innovation, Division of Mental Health and Addiction, Oslo University Hospital, Oslo, Norway; 4 Department of Psychology, University of Oslo, Oslo, Norway; 5 Department of Psychology, Norwegian University of Science and Technology, Trondheim, Norway; 6 Section for Treatment Research, Division of Mental Health and Addiction, Oslo University Hospital, Oslo, Norway; 7 Institute of Clinical Medicine, University of Oslo, Oslo, Norway; 8 Nydalen District Psychiatric Centre, Division of Mental Health and Addiction, Oslo University Hospital, Oslo, Norway; 9 Université Paris Cité, INSERM UMR-S 1144, Optimisation Thérapeutique en Neuropsychopharmacologie OTeN, Paris, France; 10 Département de Psychiatrie et de Médecine Addictologique, AP-HP, Groupe Hospitalo-Universitaire AP-HP Nord, DMU Neurosciences, Hôpital Fernand Widal, Paris, France; 11 Center for Precision Psychiatry, Division of Mental Health and Addiction, University of Oslo & Oslo University Hospital, Oslo, Norway

**Keywords:** Bipolar disorder, Mood, Nicotine use, Alcohol use, Time-series analyses, App monitoring

## Abstract

**Background:**

The prevalence of substance use disorders in bipolar disorder (BD) is high. Exploring potential interactions between mood and the use of common substances such as alcohol and nicotine may contribute to a better understanding of the mechanisms underlying such comorbidities. Digital tools now allow for continuous monitoring and data collection of both symptoms and behavior. This enables time-series analyses to explore such associations with greater precision.

**Methods:**

Thirty-two individuals in the early phases of BD registered their mood daily and their use of substances weekly in the MinDag (MyDay) app for up to 6 months. We explored temporal relationships between the use of alcohol and nicotine and the levels of depressed, elevated, irritable, and anxious mood using Vector Autoregressive Models and Granger causality tests.

**Results:**

We found indications that mood influenced alcohol- and nicotine use, and vice versa. Significant temporal relationships (Granger causality) were found in 55% (11 out of 20) of the participants for alcohol and 70% (7 out of 10) for nicotine use, and with high proportions of the variance explained by the one time-series on the other. The associations were consistent with causal effects in one or both directions, but with no adjustment for confounding.

**Conclusion:**

Our findings indicate that mood influences alcohol- and nicotine use and vice versa in individuals with BD, although caution should be taken due to the exploratory approach. Larger samples are needed to further disentangle these relationships to provide insight for better prevention and treatment of BD and comorbid substance use disorders.

## Introduction

Bipolar disorder (BD) is a mental illness characterized by repeated episodes of depression and mania or hypomania. A majority of individuals with BD may spend a considerable amount of time in affective episodes each year, with recurrence rates of both mania and depression averaging approximately one episode per year (Tondo et al. 2017). Given the recurrent nature of BD, it is vital to identify predictors of affective symptom exacerbations (Grande et al. 2016).

Excessive use of alcohol is common in individuals with BD (Blanco et al. 2017), with use often developing into abuse or dependence and with lifetime prevalence rates of 30% for alcohol use disorder (AUD) (Hunt et al. 2016). For comparison, the mean lifetime prevalence of AUD in the general population is around 9% (Glantz et al. 2020). Studies indicate that AUD can negatively impact the course of BD with increased episode recurrence and risk of rapid cycling (Rakofsky and Dunlop 2013), increased risk of suicide attempts and suicidal ideation, as well as worse social functioning (Preuss et al. 2020). AUD has also been associated with longer time until remission from affective episodes, as well as increased depressive and manic symptoms, comorbid substance use disorder (SUD), and higher impulsiveness and affective lability (Icick et al. 2021; Lagerberg et al. 2017; Pinto et al. 2020). Also, lifetime AUD has been associated with higher levels of depressive symptoms in BD patients (Cardoso et al. 2008), and in women with BD, increased consumption of alcohol appears to be associated with increases in both depressive and manic symptoms (Gordon-Smith et al. 2020). However, most research has been conducted on lifetime comorbid AUD so far (Icick et al. 2021), and little is known about the influence of use that does not meet the criteria for a use disorder on BD symptoms.

With regards to nicotine, the use has been significantly reduced in the general population over the last decades, while this is not the case for individuals with BD (Rødevand et al. 2019). Here, the lifetime prevalence rates are still as high as 46% for tobacco use disorders (TUD)/nicotine dependence (Fornaro et al. 2022). There are indications from cross-sectional studies that TUD may be associated with more lifetime mood episodes (Gross et al. 2020), and being a smoker has been associated with having more manic episodes per year of illness (Icick et al. 2017). There is also an association between smoking and suicide attempts in individuals with BD (Ducasse et al. 2015).

Given that most studies investigating the relationship between nicotine and alcohol use disorders and the course of BD are cross-sectional, there are still controversies with regards to the putative directions of causality; does substance use cause affective symptoms and syndromes, or does the presence of affective symptoms cause more substance use? Furthermore, existing longitudinal studies of the relationship between substance use and the course of BD have focused mainly on established AUD or SUDs, or have had a large proportion of participants with these disorders in their sample (Baethge et al. 2008; van Zaane et al. 2014). However, research focusing on younger samples with shorter BD illness duration and who have not yet developed AUD, is still needed. In a recent study by our group where we focused on non-pathological alcohol- and nicotine use, we found no associations between use of these substances and affective symptoms in individuals with BD without SUDs (Glastad et al. 2023). However, there may still be individual within-person interplays between the use of alcohol, nicotine and mood symptoms that were not identified due to the cross-sectional design. Smartphone-based collection of day-to-day data could be a way to explore these possible temporal relationships in a more valid and in-depth manner with new statistical methods. Previous longitudinal studies on temporal relationships between affective states and alcohol use have been based on paper-and-pencil methods for self-report and/or low resolution in the time-intervals used to investigate the interplays, both with higher risk for recall bias compared to frequent digital self-report (Baethge et al. 2008; Faurholt-Jepsen et al. 2016; van Zaane et al. 2014).

Both alcohol and nicotine exert immediate rewarding psychological effects through their effects on serotonergic and dopaminergic systems (Singh 2021; Wittenberg et al. 2020). Thus, the association between mood symptoms and use of nicotine and alcohol may be understood as substances being used as an attempt to regulate affective states, often called self-medication (Khantzian 1997). Indeed, results from a systematic review indicated that individuals with BD use alcohol to relieve distressing mood, but also to enhance euphoric mood (McDonald and Meyer 2011). However, in previous studies participants have been asked whether they use substances to alleviate symptoms, with a potential risk of bias due to a possible rationalization of a stigmatized behavior (Bolton et al. 2009; Canham et al. 2018; Pettersen et al. 2013). To our knowledge, possible self-regulation of BD symptoms with nicotine has previously not been investigated specifically.

In this study, we will investigate the temporal relationship between changes in mood (i.e. levels of mood elevation, irritability, sadness, and anxiety) and alcohol- and nicotine use over time through time-series analyses of data from app-based self-monitoring in individuals with BD. The aim is to explore whether changes in mood are followed by changes in alcohol- or nicotine use, and vice versa. We also investigate whether putative temporal relationships between alcohol use and mood differ between individuals with and without AUD, and/or the use of other substances. Since the statistical approach we have applied should be considered exploratory, we do not put forward any clear hypotheses.

## Materials and methods

### Participants

The study was conducted at the NORMENT centre (Norwegian Center for Mental Disorders Research) at Oslo University Hospital (OUS) in Oslo, Norway. The participants were recruited from the Bipolar Unit at Nydalen District Psychiatric Center (OUS). This is a catchment-area-based, out-patient specialized care unit providing treatment for patients in the early course of BD. The catchment-area represents all social strata. Inclusion criteria for participation in the current study were a diagnosis of BD type I, II or NOS (not otherwise specified), age between 18 and 65 years and willingness to use the MinDag (My Day) app to self-report mood and substance use for up to 6 months. Exclusion criteria were lack of understanding of a Scandinavian language. The total sample comprised 32 participants: 12 with BD I, 17 with BD II, and 3 with BD NOS.

### Diagnostic evaluations

Diagnostic evaluations were conducted by a medical doctor, psychiatrist, or clinical psychologist using the Structured Clinical Interview for DSM-IV axis I disorders (SCID) modules A-E or the SCID-5 Clinicians’ Version (CV). Since the DSM-5 CV only covers current disorders, the lifetime substance use disorder module of SCID-5 Research Version was added for the purpose of the study (First 2002; First et al. 2016). Diagnoses were systematically discussed in consensus meetings in the presence of experienced clinical specialists/PhDs in psychiatry and psychology.

### App-based longitudinal self-report with MinDag

The MinDag app was developed by researchers at the NORMENT centre in collaboration with the Center for Information Technology at the University of Oslo (Bjella et al. 2022). MinDag comprises six modules, and data from two of them were used for the purpose of the current study. The MinDag mood section consists of an adapted version of a subscale of the Multidimensional Assessment of Thymic States (MAThyS) with 7 items covering happiness, sadness, anger, irritability, mood elevation, anxiety and panic. The items are rated on a 7- point Likert scale from “not at all” to “to a large degree” (Henry et al. 2008), where higher scores indicate higher levels of the respective emotional states. Participants are prompted by the app to rate all mood items on a daily basis. In this study we focused on sadness, mood elevation and irritability as they represent the core mood features of BD, along with anxiety, which is a common comorbid symptom in BD.

Also, once a week, the participants were prompted by the app to register their use of alcohol, nicotine, and other substances:

1) Nicotine (cigarettes, “snus” (in Scandinavia a legal smokeless tobacco), and e-cigarettes): If the participants indicated that they had used nicotine, they were asked how many units of nicotine they had used on average each day during the last week. It was possible to register use of all three nicotine types during 1 week.

2) Alcohol: If the participants indicated that they had used alcohol, they were asked to report how many days during the last week they had been drinking, and how many units they had consumed of either wine, beer or drinks in total during the last week.

3) Other substances: If the participants indicated that they had used any of the following substances during the last week: hashish/marihuana, amphetamine/methamphetamine, cocaine, heroin, GHB/GBL, anabolic steroids, hallucinogens, or unprescribed medications, they were asked to report how many days they had used each substance during the last week.

### App-based data completeness and imputation

The participants were asked to use the app for 6 months but were informed that shorter periods were also valuable and acceptable. Cases were excluded from the analyses if they had registered data only for a period of 2 weeks or less. This was the case for nine participants, reducing the sample from N = 41 to N = 32. In this final sample, the range of weekly registrations per participant was 7–27 weeks, with a mean of 18.3 weeks. To synchronize mood and substance use intervals to enable time-series modelling, we calculated the weekly mean scores for the four mood variables, which in some cases were based on less than seven days due to sporadically missing data. If relevant data (alcohol, nicotine, or mood registrations) was missing at the start or the end of a time-series, the series was shortened accordingly. In cases of missing data in a time-series, which was the case in 102 out of a total of 633 weeks across the whole data set, values were imputed by the mean of the preceding and subsequent value of the time-series. After the imputation, the total number of weeks used in the analyses was 587. The proportion of missing data was thus 16.1%.

### Statistical analyses

The statistical analyses were conducted with the IBM Statistical Package for Social Sciences version 29 and R. A Vector Autoregression Model (VAR) was used for multivariate time-series modelling of the pairwise relationships between mood (any of the four mood items) and alcohol and mood and nicotine, implemented in the R-package *vars* (Lütkepohl 2005; Pfaff 2008). Although VAR models and time-series analyses have previously been used to investigate behavioral within-person relationships (Lorenz et al. 2020), the approach should be considered explorative and mainly as a means of generating hypotheses. In such a model, each variable is regressed on time-lagged values of itself and time-lagged values of other variables. Ordinary least squares (OLS) is used for parameter-estimation. To investigate stationarity, i.e. that the time-series’ statistical properties such as mean and variance remain constant over time, we used the Dicky-Fuller augmented test (Banerjee 1993; Said and Dickey 1984). A potential causal relationship was assessed by means of testing for “Granger-causality” (*vars*). One time-series ${y}_{1,t}$ is said to Granger-cause another time-series ${y}_{2,t}$ if predictions of ${y}_{2,t}$ based on its own history and past values of ${y}_{1,t}$ are better than predictions of ${y}_{2,t}$ based only on its own past values. As the study was explorative, i.e. with the aim to detect any potential temporal relationships between alcohol or nicotine and mood, we applied a p-value threshold for significant Granger-causality of p ≤ 0.1.

Pairwise VAR models were fitted, one with different mood variables (one at a time) and number of alcohol units (transformed), and another with different mood variables and number of nicotine units. Two lags were included for a flexible model, and to limit the number of parameters. Each VAR model can be expressed as:

1 $$\begin{aligned} {y}_{1,t}&={\alpha }_{0}+{\alpha }_{1}{y}_{1,t-1}+{\alpha }_{2}{y}_{1,t-2}+{\alpha }_{3}{y}_{2,t-1}+{\alpha }_{4}{y}_{2,t-2}+{\varepsilon }_{1,t} \\ {y}_{2,t}&={\beta }_{0}+{\beta }_{1}{y}_{1,t-1}+{\beta }_{2}{y}_{1,t-2}+{\beta }_{3}{y}_{2,t-1}+{\beta }_{4}{y}_{2,t-2}+{\varepsilon }_{2,t} \end{aligned}$$

In case of non-stationarity, a time series with the differences between the lagged values was formed (${D}_{1,t}={y}_{1,t+1}-{y}_{1,t}$) and used instead (iterated if the difference series was non-stationary). Impulse response and forecast error variance decomposition (FEVD) was calculated to assess the impact of one time-series on the other (*vars*). The impulse response illustrates how the dynamic system reacts to a “shock” (equal to 1 standard deviation (Std)) in one of the error terms, and can be used to interpret the impact of e.g. mood variables on alcohol use, or vice versa. This is represented by a plot of the forecast of each variable in the fitted model with corresponding bootstrapped 95% confidence intervals. The FEVD expresses how much of the forecast (prediction) error in one series, for a certain number of steps ahead, that can be attributed to the other series. A simple interpretation is that it corresponds to the proportion of explained variance due to the other series.

A separate VAR model was fitted for each participant. Inference across participants was therefore a challenge. The total number of VAR models were summarized to compare participants descriptively without testing and should be viewed as exploratory analyses. One participant had a stationary time-series, but the analyses yielded non-valid results and was therefore not included.

## Results

### Demographics and clinical characteristics

The demographic and clinical characteristics of the total sample are presented in Table 1. The number of units used of alcohol and nicotine are based on app-based self-report and presented for participants who reported any use of the specific substance: 32 (100%) for alcohol and 19 (59.4%) for nicotine.

**Table 1:** Demographic and clinical characteristics of the sample (N = 32)

Characteristics	Total
Female sex, *n* (%)	19 (59.4)
Age, years, mean (Std)	33.28 (10.1)
Range, years	21–57
Data collection period, weeks, mean (Std)	18.3 (6.24)
Range, weeks	7–27
Missing data, weeks (%)	102 (16.1)
BD subtype, *n* (%)	
BD I	12 (37.5)
BD II	17 (53.1)
BD NOS	3 (9.4)
Comorbid disorders,* n* (%)	13 (40.6)
Alcohol use disorder, current, *n* (%)	2 (6.3)
Alcohol use disorder, lifetime (%)	2 (6.3)
Illicit^a^ substance use disorder, current,* n* (%)	1 (3.1)
Illicit^a^ substance use disorder, lifetime,* n* (%)	2 (6.3)
Anxiety disorder^b^,* n* (%)	8 (25.0)
Nicotine use, *n* (%)	19 (59.4)
Nicotine units per week, mean (Std)	9.64 (10.3)
Range, units per week	0–130
Alcohol use, *n* (%)	32 (100)
Alcohol units per week, mean (Std)	7.45 (8.2)
Range, units per week	0–44
Any illicit substance use, *n* (%)	5 (15.6)

BD: Bipolar Disorder, Std: Standard Deviation; ^a^includes the following Use Disorders; Cannabis, Cocaine, MDMA, and Hallucinogens. ^b^includes Social Phobia, Generalized Anxiety Disorder, Panic Disorder, Agoraphobia and Post Traumatic Stress Disorder

### Alcohol (transformed) and mood

Twenty participants (62.5%) of those who reported alcohol use had stationary alcohol series, and all of these had at least one stationary mood series. Of these 20, 11 (55%) had Granger causality tests representing significant effects in one or both directions between a mood variable and alcohol use (Table 2).

**Table 2:** Significant Granger causality between mood and alcohol units per week (transformed with square root)

Mood	Number of participants
Mood → Alcohol	6 (id: 5, 14, 20, 30, 31, 36)
Alcohol → Mood	3 (id: 2, 13, 27)
Mood ↔ Alcohol	2 (id: 22, 25)

One of the participants with stationary alcohol series had a current AUD. The time-series of this participant showed a significant Granger-causal relationship from alcohol use to mood. Two participants with stationary time-series had previous SUDs involving several other substances than alcohol and nicotine. Both had significant Granger causality between mood and both alcohol- and nicotine use. Furthermore, four participants, all with stationary time-series on either alcohol use, nicotine use, or both, reported use of other substances during the study period. Only one of them, the same participant who also had an AUD, had a significant Granger-causal relationship from alcohol use to mood. Of the five participants who had a previous SUD involving other substances or a current or previous AUD, only one reported use of other substances during the study period.

**Fig. 1:**
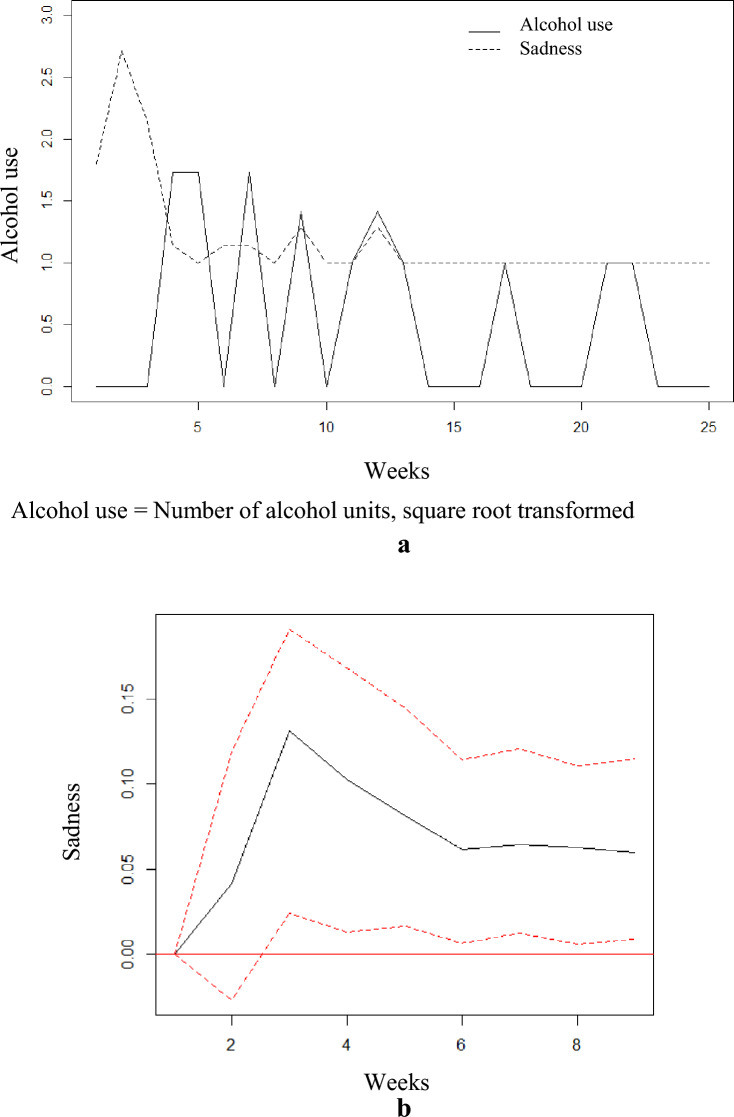
**a** Time course of alcohol units per week (transformed) and sadness for ID = 25. Alcohol use = Number of alcohol units, square root transformed. **b** Impulse response function in sadness, from a “shock” (= 1 Std) in alcohol units, ID = 25. The red dotted lines represent 95% bootstrap confidence intervals, with 200 runs" should be placed under Figure 1b

For illustration, the raw time-series data for participant with ID = 25 is plotted in Fig. 1a, with alcohol use (units per week, square root transformed) as the solid line, and mean sadness per week as the dotted line.

For this participant, alcohol use was shown to significantly Granger cause sadness, which was supported by the impulse response function (Fig. 1b). A sudden short-term increase (“shock”) in alcohol use led to a significant long-term increase in sadness with the 95% bootstrap confidence interval not covering 0 from three to nine weeks. Furthermore, the variance decomposition showed that a large amount of the variation in mood, in this case sadness, was explained by alcohol use (as shown in Table 3). A maximum of 23% of the variance in this participants sadness was explained by alcohol consumption, over a period of 10 weeks.

**Table 3:** Forecast error variance decomposition (FEVD) for participants with significant Granger causality between mood and alcohol use

Granger Causality	Lag 1^a^	Lag 2	Lag 3	Lag 4	Lag 5	Lag 6	Lag 7	Lag 8	Lag 9	Lag 10	p-value
Mood Granger causes alcohol use											
ID 5	0.02	0.01	0.14	0.11	0.15	0.12	0.13	0.12	0.12	0.11	.014
ID 14	0.01	0.03	0.22	0.27	0.28	0.34	0.36	0.36	0.37	0.38	.003
ID 20	0.02	0.24	0.34	0.32	0.32	0.33	0.33	0.33	0.33	0.33	.009
ID 22^*^	0.05	0.08	0.31	0.36	0.36	0.37	0.37	0.39	0.39	0.39	.043
ID 25^*^	0.01	0.06	0.10	0.12	0.13	0.14	0.14	0.15	0.15	0.15	.002
ID 30	0.04	0.16	0.35	0.43	0.43	0.43	0.44	0.44	0.44	0.44	.048
ID 31	0.04	0.06	0.18	0.16	0.15	0.16	0.16	0.16	0.16	0.15	.022
ID 36	0.03	0.33	0.32	0.34	0.34	0.36	0.36	0.37	0.37	0.38	.001
Mean	0.03	0.12	0.25	0.26	0.27	0.28	0.29	0.29	0.29	0.29	
Alcohol use Granger causes mood											
ID 2	0.00	0.18	0.32	0.32	0.32	0.32	0.33	0.33	0.33	0.33	.017
ID 13	0.00	0.06	0.06	0.07	0.06	0.06	0.06	0.06	0.05	0.05	.091
ID 22^*^	0.00	0.03	0.31	0.42	0.42	0.48	0.49	0.49	0.50	0.51	.015
ID 25^*^	0.00	0.02	0.15	0.20	0.21	0.22	0.22	0.23	0.23	0.23	.028
ID 27	0.00	0.01	0.15	0.26	0.30	0.29	0.29	0.30	0.30	0.29	.054
Mean	0.00	0.06	0.20	0.25	0.26	0.27	0.28	0.28	0.28	0.28	

The table shows significant Granger causality between mood and alcohol use over a period of ten weeks. For patients with significant Granger causality between more than one Mood variable, only one of the FEVDs is presented. A simple interpretation of the FEVD is that it corresponds to the proportion of explained variance due to the other time-series. ^a^Lag numbers correspond to weeks, ^*^The participant has a bidirectional relationship

Table 3 shows the forecast error variance decomposition (FEVD) for participants with significant Granger causality between mood and alcohol use. Averaged over the eight participants with significant effects in the same direction i.e. mood to alcohol including the participants with a bidirectional relationship, 29% of the variance, i.e. forecast error variance decomposition as a function of time, in alcohol was explained by a mood variable at 10 weeks, with a range from 11 to 44%. For the five participants with significant effects from alcohol to mood including the participants with a bidirectional relationship, 28% of the variance in mood was explained by alcohol at 10 weeks, with a range from 5 to 51%.

### Nicotine units (cigarettes, snus) and mood

Ten participants (53%) of those who reported nicotine use, had stationary time series of nicotine use, and of these, seven (70%) had significant tests for Granger causality in one or both directions between nicotine use and mood (Table 4).

**Table 4:** Significant Granger causality between mood and nicotine units per week by identification number

Mood	Number of participants
Mood → Nicotine	4 (id: 2, 12, 31, 36)
Nicotine → Mood	3 (id: 5, 22, 25)
Mood ↔ Nicotine	0

**Fig. 2:**
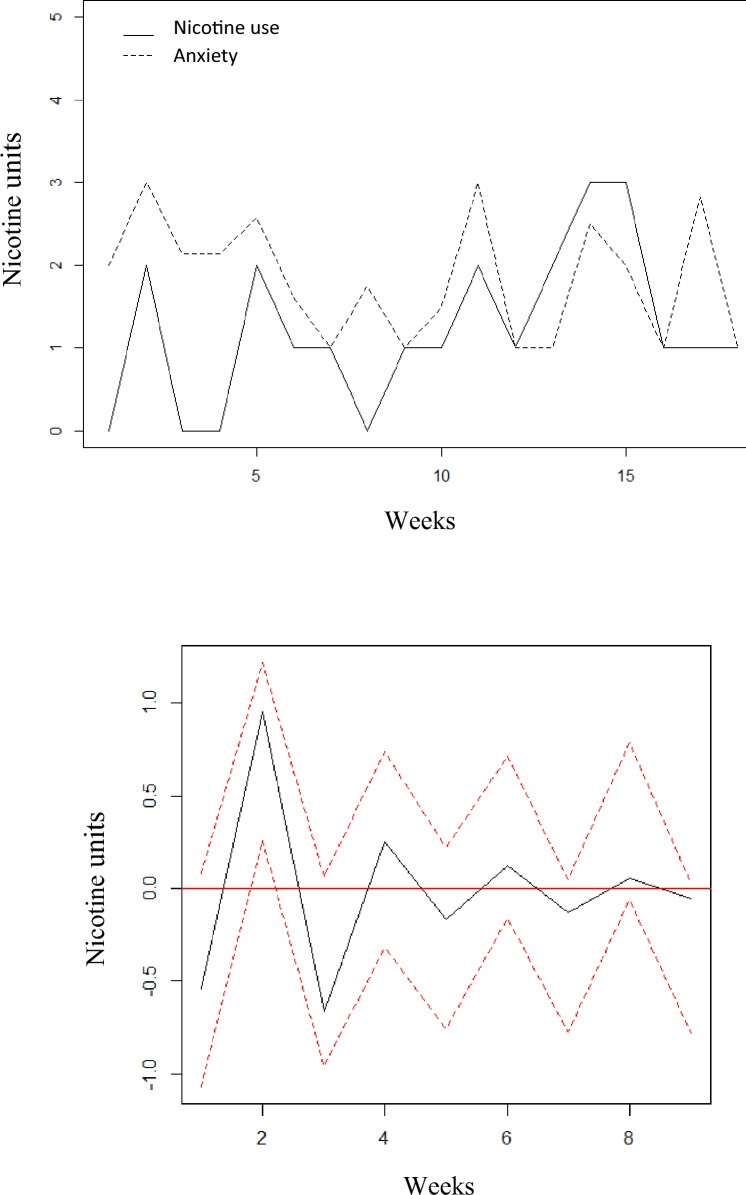
**a** Time course of nicotine units per week and anxiety for ID = 36. **b** Impulse response function in nicotine units, from a “shock” (= 1 Std) in anxiety, ID = 36. The red dotted lines represent 95% bootstrap confidence intervals, with 200 runs

For illustration, the raw time series for anxiety and total number of nicotine units per week for participant with ID = 36 is shown in Fig. 2a.

Anxiety was found to significantly Granger cause nicotine use, and this was supported in the impulse response function (Fig. 2b). A “shock” in anxiety led to a significant short-lived change in nicotine that disappeared after 3 weeks. Nicotine use explained a maximum of 65% of the variance in mood over a period of 10 weeks (Table 5).

**Table 5:** Forecast error variance decomposition (FEVD) for patients with significant Granger causality between mood and nicotine use

Granger Causality	Lag 1^a^	Lag 2	Lag 3	Lag 4	Lag 5	Lag 6	Lag 7	Lag 8	Lag 9	Lag 10	p-value
Mood Granger causes nicotine use											
ID 2	0.01	0.01	0.13	0.23	0.24	0.25	0.27	0.27	0.27	0.27	.028
ID 12	0.16	0.18	0.29	0.30	0.31	0.31	0.31	0.31	0.31	0.31	.080
ID 31	0.08	0.19	0.14	0.13	0.14	0.13	0.14	0.13	0.13	0.13	.051
ID 36	0.26	0.57	0.64	0.65	0.65	0.65	0.65	0.65	0.65	0.65	.016
Mean	0.13	0.24	0.30	0.33	0.34	0.34	0.34	0.34	0.34	0.34	
Nicotine use Granger causes mood											
ID 5	0.00	0.12	0.40	0.43	0.45	0.48	0.48	0.48	0.48	0.48	.032
ID 22	0.00	0.07	0.21	0.33	0.39	0.41	0.41	0.41	0.41	0.41	.077
ID 25	0.00	0.02	0.08	0.08	0.08	0.09	0.09	0.09	0.09	0.09	.097
Mean	0.00	0.07	0.23	0.28	0.31	0.33	0.33	0.33	0.33	0.33	

The table shows significant Granger causality between mood and nicotine use over a period of ten weeks. A simple interpretation of the FEVD is that it corresponds to the proportion of explained variance due to the other time-series. ^a^Lag numbers correspond to weeks

Averaged over the four participants with significant effects from mood to nicotine use, 34% of the variance in nicotine use was explained by mood at 10 weeks, ranging from 13 to 65%. Averaged over the three participants with significant effects from nicotine use to mood, 33% of the variance in mood was explained by nicotine use at 10 weeks, ranging from 9 to 48%.

## Discussion

In this study, we examined temporal relationships between weekly self-reported mood and alcohol- and nicotine use in a sample of individuals with BD. We found associations between mood and subsequent alcohol- and nicotine use and vice versa. Significant temporal relationships (Granger causality) was found in 55% (11 out of 20) of the participants for alcohol use and 70% (7 out of 10) for nicotine use. The results also indicate that a high proportion of the variance in one variable, e.g. nicotine use, was explained by the other variable, e.g. mood. The associations were consistent with causal effects in one or both directions, although this should be interpreted with caution as the analyses are exploratory. To our knowledge, this is the first published study to date to explore the temporal relationship between nicotine use and mood in BD using app-based longitudinal self-report.

We identified several cases where a change in mood appeared to influence alcohol use during the subsequent weeks and found that a substantial proportion (29%) of the variance in alcohol use was explained by a change in mood. This could suggest that some individuals with BD self-regulate, i.e. attempt to alleviate mood symptoms by increasing their alcohol intake. This is in line with a previous study reporting that poorer emotional self-regulation is associated with increased risk of alcohol- and other substance use disorders in individuals with BD (Wilens et al. 2013). Other studies investigating whether individuals with BD use substances to alleviate mood symptoms have mainly asked the patients directly about this relationship (Bolton et al. 2009; Canham et al. 2018). With this methodology there is considerable risk of response bias due to the tendency to rationalize stigmatized behavior. Although still not eliminated, this risk is likely to be reduced when substance use and mood are independently reported in parallel over time.

We also found that in some cases, changes in alcohol use appeared to have a long-term influence on mood. For instance, as in one participant, a sudden change in alcohol use was followed by a significant change in sadness that persisted for several weeks. Although there were cases where changes in alcohol use were followed by changes in mood, these were of somewhat lower frequency compared to cases where mood appeared to influence alcohol use. As with the opposite relationship, a relatively large proportion of the variance in mood was explained by a preceding change in alcohol use. Although speculative, one may hypothesize that a subgroup of individuals with BD are particularly sensitive to the effects of alcohol on mood. This contrasts the results from our previous study where no significant associations between the levels of alcohol- or nicotine use and the levels of manic- or depressive symptoms were found (Glastad et al. 2023). Although the study had a large and representative sample size, it was cross-sectional in design and thus interactions over time could not be explored. The results from the current study also contrasts a previous study where the effects of alcohol use on changes in affective states were limited but mainly related to more euthymia (van Zaane et al. 2014). This study is, however, not directly comparable to the current, as it investigated transitions between affective states, i.e. depression, euthymia etc., and not changes in mood states independently from affective syndromes, as in our study. Our current findings may, however, be more in line with a few previous studies indicating that alcohol use and AUD increase the risk of both affective symptoms and episodes (Cardoso et al. 2008; Gordon-Smith et al. 2020).

Two cases showed a bidirectional relationship between mood and use of alcohol. Possibly, in some cases, the relationship between mood and alcohol use may be mutually reinforcing, causing a vicious circle of escalating mood disturbance and alcohol use over time. Such mechanisms may contribute both to the high risk of AUD in BD and to the more destabilized illness course commonly seen in BD with comorbid AUD (Rakofsky and Dunlop 2013).

As there was only one participant in our sample with a current AUD who presented with significant temporal relationships from alcohol use to mood, comparison with non-AUD participants was not justified. Four participants reported use of other substances than alcohol and nicotine during the study period, all of which had time-series that were amenable to analysis on either alcohol use, nicotine use, or both. Only one of these participants, the same who had an AUD, had a significant temporal relationship from alcohol use to mood. Further studies on larger samples are needed to investigate interactions between mood and substance use across relevant subgroups, including individuals with current and remitted SUDs. Such knowledge may shed light on differences in mechanisms between those who succeed in recovering from their SUD and those who continue their use.

We found that changes in mood was followed by changes in the level of nicotine use in some participants. Further, changes in mood appeared to have a long-term influence on nicotine use in some cases, and a substantial proportion of the variance (34%) in nicotine use was explained by a change in mood. Thus, our findings may indicate that nicotine is being used to self-regulate mood by some individuals with BD, in line with a study reporting a relationship between deficits in emotional self-regulation and nicotine use in individuals with BD (Wilens et al. 2013). Of note, we also found the opposite relationship, namely that changes in nicotine use in some cases were followed by changes in mood.

Except from our own previous cross-sectional study, where no associations between nicotine use and affective symptoms were found (Glastad et al. 2023), we are not aware of any other studies on BD where the temporal relationship between mood and nicotine use has been explored. Our preliminary findings, taken together with findings from previous cross-sectional studies where nicotine use has been associated with more manic episodes per year of illness (Icick et al. 2017), lifetime mood episodes (Gross et al. 2020), and suicide attempts (Ducasse et al. 2015), indicate that nicotine use may be of relevance for mood and mood regulation in BD. Thus, further longitudinal studies should investigate the impact of nicotine use on mood, and vice versa, in BD. Future research should include longitudinal neuroimaging studies investigating the impact of nicotine on brain activity in regions related to mood. Furthermore, investigating whether specific genes or epigenetic modifications make individuals with BD more susceptible to nicotine dependence, or influence how nicotine affects mood, are important future avenues. A better understanding of these mechanisms could pave the way for more efficient interventions targeting nicotine use in BD, which although speculative, may also be beneficial for the course of the BD itself.

The use of alcohol and nicotine was relatively low in the current sample. Still, a fairly large number of cases presented with significant relationships between mood and alcohol- and/or nicotine use. Again, with the necessary interpretative precautions, this may indicate that even smaller amounts or changes in use of these substances can influence mood or vice versa.

## Limitations and strengths

The current study is explorative and although several significant temporal relationships between the variables of interest were found, they should be interpreted with caution. The main limitation of the current study was the small sample size, which prevented subgroup analyses with different levels and patterns of substance use. The small sample size was in part a consequence of the requirement of stationarity in the data of VAR analyses. Still, most cases met the requirements after differencing the series and could be kept in the analyses. Despite the limited sample size, several significant relationships were identified, although we were not able to differentiate between specific mood items. This would have been of interest and should be explored in future studies. Also, we did not have information about whether the participants experienced affective episodes during the registration period. Furthermore, the level of missing data was sub-optimal, and imputation was necessary to enable VAR analyses, with potential risks of inducing bias in the data. However, the proportion of missing data was comparable to other studies based on digital self-monitoring (Faurholt-Jepsen et al. 2016). Also, nicotine use was relatively low and stable across most of the time series, while alcohol use had a more varied pattern. In addition, we applied a liberal threshold for statistical significance and performed many statistical tests without correcting for multiple testing. This was still considered an adequate approach as the study was explorative, taking advantage of rich longitudinal data but applying statistical methods that have been developed for other purposes. Thus, there was risk of inflation of type I error, and further larger studies are warranted to validate our findings. Also, no control for confounding was performed.

The study also has several strengths. The main strength is the repeated real-time monitoring of mood and alcohol and nicotine use which yields high-resolution longitudinal data where effects between variables over time can be explored. The mean explained variance among the participants with significant associations is large. The findings are descriptive but can be consistent with causal effects. Also, VAR models are a powerful statistical tool for analysis of multiple parallel time-series and exploration of causal relationships. Lastly, our sample of individuals with BD is catchment-area-based and are included in the early course of BD, thus likely to be representative for the early phase BD population.

## Conclusion

We found significant temporal relationships between changes in mood and levels of alcohol- and nicotine use in individuals with BD, which provides a basis for hypothesizing that alcohol and nicotine use influence mood levels in BD and vice versa. Future studies should focus on further disentangling these relationships, focusing on differentiating between mood items and individual risk of substance use escalation. Although there are limitations to the clinical generalizability of the results based on the explorative nature of the analyses, they may contribute to a better understanding of the mechanisms of substance use disorder development in BD, which in turn may enhance its prevention and treatment, with potential benefit for the patients.

## Data Availability

The dataset used in the current study are available from the corresponding author on reasonable request.

## Abbreviations

AUD: Alcohol use disorder
BD: Bipolar disorder
CV: Clinicians’ Version
FEVD: Forecast error variance decomposition
MAThyS: Multidimensional Assessment of Thymic States
NORMENT: Norwegian Center for Mental Disorders Research
NOS: Not otherwise specified
OLS: Ordinary least squares
OUS: Oslo University Hospital
SCID: Structured Clinical Interview for DSM-IV axis I disorders
Std: Standard deviation
SUD: Substance use disorder
TUD: Tobacco use disorder
VAR: Vector autoregression model
